# Impact of Psychotherapy for Children and Adolescents with Anxiety Disorders on Global and Domain-Specific Functioning: A Systematic Review and Meta-analysis

**DOI:** 10.1007/s10567-022-00402-7

**Published:** 2022-07-07

**Authors:** Sophie J. Dickson, Rebecca-Lee Kuhnert, Cassie H. Lavell, Ronald M. Rapee

**Affiliations:** Centre for Emotional Health, School of Psychological Sciences, Macquarie University, Sydney, NSW 2109 Australia

**Keywords:** Functioning, Psychotherapy, Anxiety, Children, Adolescents, Meta-analysis

## Abstract

A substantial empirical base supports the use of psychotherapy to alleviate anxiety symptoms and diagnoses in children and adolescents. However, focusing only on symptom or diagnostic reduction provides an incomplete picture of clinically meaningful efficacy given that anxiety disorders in this age group are integrally associated with problems in functioning. A systematic review and meta-analysis (*N* studies = 40, *N* participants = 3094) evaluating the impacts of psychotherapy for anxiety was conducted on the following outcomes: global functioning, social functioning, academic functioning, and school attendance. Randomised controlled trials with a passive control condition, a child and/or adolescent sample (7–17 years) with a primary anxiety diagnosis, and receiving anxiety-focused psychotherapy were eligible for inclusion if they reported suitable outcome data. Results from the meta-analysis indicated that from pre- to post-treatment, psychotherapy led to significant improvements in global functioning according to clinician (*d* = 1.55), parent (*d* = 0.67), and child (*d* = 0.31) reports and on social functioning according to parent (*d* = 0.51), but not child (*d* = 0.31) reports. The qualitative review provided preliminary support psychotherapy’s efficacy in increasing family functioning and school attendance, but not so much in enhancing academic performance. These results indicate that psychotherapy improves daily functioning in anxious children and adolescents. The study also highlighted the limited attention paid to measures of functioning in the empirical literature on treatment of childhood anxiety.

Trial Registry: This study is registered with PROSPERO under the identification number CRD42021246565.

**Supplementary Information:**

The online version contains supplementary material available at 10.1007/s10567-022-00402-7.

Significant attention has been given to the evaluation of psychological treatments for anxiety disorders in childhood and adolescence over recent decades. Predominantly, researchers examining the efficacy of anxiety treatments for children and adolescents have evaluated outcomes based on symptom and/or diagnostic improvements. However, focusing only on symptoms and diagnoses provides an incomplete picture of clinically meaningful efficacy given that anxiety disorders in this age group are integrally associated with problems in functioning (Becker et al., 2011; Rapee et al., 2012). In childhood and adolescence, the term functioning refers to an individual’s ability to engage in routine activities and adapt to role expectations across multiple domains of living: at home, at school, or with peers (Hoagwood et al., 2012). Functioning may also be conceptualised globally, whereby unidimensional measures of functional impairment yield a summary score for individuals across multiple settings. Treatment studies for youth anxiety report a parallel relationship between global functioning and symptoms, that is, as anxiety improves, functioning scores increase (e.g. Holmes et al., 2014; Perrin et al., 2019). Measures of global functioning designed specifically for use with anxious young people include the Child Anxiety Life Interference Scale (CALIS; Lyneham et al., 2013) and the Child Anxiety Impact Scale (CAIS; Langley et al., 2004). The simplicity of a unidimensional rating facilitates comparisons of anxious young people with normative peers and with peers with different disorders, as well as the convenience of evaluating changes in functioning over time (Goldman et al., 1992). A drawback of this approach is that a single rating does not reveal which specific areas of functioning are most affected by the disorder and hence constitute the most appropriate treatment targets (Winters et al., 2005).

An association between anxiety disorders and specific domains of impaired functioning has been widely documented in child and adolescent samples (Settipani & Kendall, 2013), including impairment in peer relationships, academic functioning, and family relationships. Compared to their non-anxious peers, young people with anxiety disorders experience more peer problems, including decreased peer acceptance (Flanagan et al., 2008), more peer victimisation (Crawford & Manassis, 2011), fewer close friends (Ladd, 1990), and poorer relationship quality (Borowski et al., 2018). Given the importance of the peer group for development and well-being, anxiety-related impairments in social functioning represent a considerable risk for negative outcomes across several areas of life (Kingery et al., 2010; Ryan, 2001).

In addition to impact on peer relationships, anxiety can have an especially salient impact on academic functioning. Potential sources of anxiety at school include *places* (e.g. the playground or classroom), *people* (e.g. staff or peers), and *performance* (e.g. tests, recitals, or public speaking), all of which may result in poorer school attendance by way of negative reinforcement (Kearney, 2008). It is not surprising, then, that poorer school attendance appears to be a particularly common consequence of paediatric anxiety disorders. One national survey showed that young people with anxiety disorders averaged 12 days absent from school within the previous 12 months due to anxiety symptoms—greater than the number of days missed by children and adolescents with attention-deficit/hyperactivity disorder and conduct disorders (Lawrence et al., 2015). In addition to school attendance, elevated anxiety predicts concurrent and long-term academic impairment (Grover et al., 2007; Mychailyszyn et al., 2010; Nail et al., 2015). For example, young people with anxiety disorders have been shown to fall gradually further behind the rest of their cohort on literacy and numeracy from grade 3 to grade 9 (Goodsell et al., 2017).

There are also clear associations between anxiety disorders and impaired family functioning (Hudson et al., 2009; Kashani et al., 1990; Lawrence et al., 2015). According to the 2015 Australian Child and Adolescent Survey of Mental Health, 19.2% of children and adolescents with *any* anxiety disorder indicated severe impairment in family life. This proportion was as high as 30.9% in children and adolescents with generalised anxiety disorder (Lawrence et al., 2015).

The prioritisation of symptoms and diagnoses over functioning may be a by-product of treatment administration under the strictly controlled conditions of efficacy trials, which prioritise the assessment of symptoms to maximise internal validity (Becker et al., 2011). Alternatively, researchers may overlook measures of functioning if they believe psychiatric symptoms and their associated impairment to be strongly and positively correlated (McKnight et al., 2016). If symptoms and functional impairment are perfectly correlated, then information acquired through impairment measurements is redundant because it contributes no further information to what is already available through symptoms and diagnoses (Rapee et al., 2012). However, if the two constructs can be demonstrated to be separable dimensions, data from both would provide a more complete picture of psychosocial recovery. Empirical evidence demonstrates only weak-to-moderate correlations between symptoms and impairment (Allen et al., 2010; Gordon et al., 2006; McKnight et al., 2016; Storch et al., 2010), indicating distinction between the constructs. Hence, treatment studies that overlook impairment may over- or under-estimate the strength of the evidence (Becker et al., 2011). For example, prevalence estimates change dramatically whether based purely on assessment of symptoms or when also taking impairment into account (Rapee et al., 2012). This criticism is slightly less applicable in reviews of diagnostic remission because diagnosis is intended to be determined by the confluence of symptomatic criteria *and* functional impairment. However, most DSM-5 and ICD-11 diagnoses require endorsement of clinically significant impairment *or* distress. Further, functional impairment is frequently measured broadly and/or imprecisely or not measured at all (McKnight et al., 2016). This may be due to the ambiguity of what is considered “clinically significant” impairment or distress. For example, the duration of distress/impairment to be considered “clinically significant” is rarely described (Spitzer & Wakefield, 1999). As a result, whilst making a diagnostic decision, functional impairment is commonly viewed as a mere covariate of interest, and symptoms that do not entail impairment are typically prioritised (Cuijpers et al., 2014). Perhaps most importantly, measures of functioning and impairment are of particular relevance to consumers. Impairment in daily functioning, more than symptoms, causes distress in anxious young people and is a significant motivator for help-seeking (Becker et al., 2011; Sanchez et al., 2019).

Despite these arguments, psychotherapy for anxiety disorders in childhood and adolescence has been evaluated primarily via symptom reduction and diagnostic remission (Ishikawa et al., 2007; James et al., 2020; Kreuze et al., 2018; Perihan et al., 2020; Reynolds et al., 2012; Scaini et al., 2016; Vigerland et al., 2016; Wang et al., 2017; Warwick et al., 2017). Reviews indicate significant, moderate-to-large effect-sized improvements in diagnostic remission and reductions in symptoms following empirically validated intervention. For example, the most recent Cochrane review reported that anxiety-focused cognitive behaviour therapy (CBT) was significantly more effective than waitlist/no treatment at reducing parent- and child-reported anxiety symptoms with moderate effect sizes of *d* = − 0.70 (95% CI − 0.90, − 0.51, Z = 7.01, *p* =  < 0.001, *N* = 2137) and − 0.67 (95% CI [− 0.47, − 0.88], Z = 6.36, *p* =  < 0.001, *N* = 2831), respectively (James et al., 2020). However, very few reviews have considered the efficacy of psychotherapy for anxiety based on functional changes.

To date, only two meta-analyses have examined the impacts of psychotherapy for paediatric anxiety disorders and expanded the outcomes of interest beyond symptom/diagnostic reduction to include measures of global functioning (James et al., 2020; Kreuze et al., 2018). As noted above, the Cochrane review by James et al. (2020) reported primarily on symptom and diagnostic improvement. However, a secondary analysis evaluated the impact of CBT on improvements in clinician-reported global functioning compared with waitlist/no treatment controls and reported a large effect (*d* = 1.03 [0.68, 1.38], Z = 5.83, *p* < 0.001; *N* = 557). The only review to date that has specifically evaluated the impact of CBT for anxiety in children and adolescents according to measures of functioning reported a large effect size improvement when combining parent, child, and clinician reports (− 1.25 [− 1.59, − 0.90], Z = 7.10, *p* < 0.001; *N* = 1234) (Kreuze et al., 2018). The authors also evaluated the specific impact of CBT against controls on social functioning. This comparison comprised six studies and yielded a non-significant effect for CBT on social functioning as reported by the parent and/or the child combined (− 0.17 [− 0.45, 0.12], Z = 1.13, *p* = 0.26). Combining data from multiple reporters will likely increase heterogeneity and may miss potentially important information due to the commonly reported low-to-moderate inter-rater agreement between parents and children regarding anxiety symptoms and impairment (Choudhury et al., 2003; Comer & Kendall, 2004; Grills & Ollendick, 2002; Rapee et al., 1994; Popp et al., 2017; Weems et al., 2011). According to De Los Reyes and Kazdin (2005) reporters disagree because of differing attributions for what causes the problem and differing perspectives on whether or which problem requires treatment. Clinical researchers must reconcile these discrepancies in a way that gives due regard to all attributions and perspectives.

Given the increased recognition of the importance of functioning in treatment of anxiety in recent years (Creswell et al., 2021), more studies are expected to have been published since the search conducted by Kreuze and colleagues in July 2016 (Kreuze et al., 2018). Furthermore, there have been comparatively few studies on the effects of treatments other than CBT variants; in fact, the two recently published reviews specifically excluded non-CBT trials (James et al., 2020; Kreuze et al., 2018). Given the potential for other psychotherapies to influence clinical practice and service development (Reynolds et al., 2012), the scope of evidence summaries should be expanded to encompass any psychological treatment for which there is evidence.

It may be assumed that broad treatments for paediatric anxiety will result in functional gains, but the evidence base is currently limited in comparison to the substantial number of studies of psychotherapy for symptom/diagnostic outcomes. Due to the critical need to establish higher standards of evidence for anxiety-focused psychotherapies in this age group, it is important to examine the efficacy of subgroups that may moderate treatment impact estimates for functional outcomes. In particular, given the frequency with which treatments are delivered either in individual or group format and also the growing evidence base for online treatments, evaluating treatment moderation by format and intensity would be valuable. These subgroups have not been investigated at the meta-analytic level, leaving unanswered questions concerning the benefits of psychotherapy for functioning according to delivery format (individual vs group therapy) and the therapy intensity (traditional therapy vs low-intensity therapy). Symptom-based meta-analyses examining delivery format and even therapy intensity have often failed to demonstrate marked differences (e.g. Ewing et al., 2015; Ishikawa et al., 2007; James et al., 2020; Vigerland et al., 2016) but the same may not be true when evaluating functioning as an outcome.

This meta-analysis therefore had three objectives. The first was to determine the overall efficacy of psychotherapy in enhancing (a) social functioning, (b) school-related functioning, and (c) global functioning in children and adolescents with anxiety disorders. It should be noted that there were insufficient studies evaluating other domains of functioning to allow meta-analysis. The second objective was to conduct subgroup analyses to examine potential moderators of these outcomes by delivery format and therapy intensity. A final objective was to analyse and report outcomes separately based on the type of reporter used (child, parent, and clinician).

## Method

The approach recommended by the Preferred Reporting Items for Systematic Reviews and Meta-analysis (PRISMA; Page et al., 2021) was used to carry out this meta-analysis.

### Eligibility Criteria

#### Types of Participants

Randomised controlled trials of children and adolescents (mean age 7–17 years) who had a primary diagnosis of an anxiety disorder or obsessive–compulsive disorder (OCD) were eligible for inclusion in the analysis. Studies were eligible if the mean age of the sample fell between 7 and 17 years, regardless of whether some participants fell outside this range. Anxiety or OCD included any recognised clinical diagnosis of an anxiety disorder or OCD as defined by the DSM-5, ICD-11, or similar standard guidelines. Whilst OCD is no longer considered an anxiety disorder within the nomenclature of the DSM-5 (American Psychiatric Association, 2013), participants with a primary diagnosis of OCD were eligible for inclusion in the current study due to the inclusion of OCD as an anxiety disorder in previous versions of the DSM and the strong comorbidity and overlap between OCD and anxiety disorders. Past reviews have established the viability of conducting meta-analysis across anxiety disorders and OCD (Ale et al., 2015; Reynolds et al., 2012).

#### Types of Interventions

All treatments incorporating a form of psychotherapy where the specific focus was to reduce anxiety were considered for inclusion. Psychotherapy could be delivered in various formats, for variable lengths of time, individually or in groups, and with varying therapist contact time.

#### Comparator Interventions

To be included, a trial had to have a passive comparator group that was a waitlist control, no treatment, treatment-as-usual, or placebo (either placebo therapy or pill placebo).

#### Types of Outcome Measures

To be included, a trial had to offer pre- and post-treatment outcome data on continuous scales relevant to at least one of the following domains of functioning: social functioning, academic performance, school attendance, family functioning, or global functioning. Randomised controlled trials (RCTs) that reported a composite summary of quality of life were excluded on the basis that functioning and quality of life are related, but empirically distinct constructs (Rapee et al., 2012). Many studies in the field include a clinician severity rating (CSR). This measure, which typically comes from diagnostic interview, is usually based on an amalgam of impairment with symptom severity (Creswell et al., 2021). Due to this confound, it was decided that CSRs would be excluded as a measure of global functioning. Because of the vast existing literature, trials were only included if the published findings met the criteria; studies that would have required further data from authors were omitted. Only peer-reviewed research published in English was considered due to a lack of resources for translation. Whilst it has been suggested that excluding trials reported in languages other than English might introduce bias and reduce the precision of treatment impact estimates, this has not been supported empirically (Jüni et al., 2002; Morrison et al., 2012). Included studies were not subject to any time limit.

### Information Sources

In late November 2021, electronic database searches on PsycINFO, Medline (Ovid), Scopus, Academic Search Premier, and Web of Science were used to identify studies for inclusion in the meta-analysis. To uncover additional studies missed by the electronic searches, a backwards snowball search was also conducted in the reference lists of reviews of similar topics.

### Search Strategy

Electronic searches were conducted using PsycINFO, Medline, Scopus, Academic Search Premier, and Web of Science. When the database permitted, studies were limited to those published in English language and in peer-reviewed journals, as per the eligibility criteria. Other than those set by the database, there were no date restrictions. A text search was conducted for key terms, taking into consideration plurals (e.g. “adolescent/s/ce”), variant spellings (e.g. “generalised/generalized” anxiety disorder), and terms used interchangeably in past papers (e.g. “functioning/impairment”). A full catalogue of search terms can be found in Appendix A.^1^

### Selection Process

The titles and abstracts of articles generated by the search procedure were screened by two independent coders to determine their eligibility for this meta-analysis. Conflicts were resolved through consultation with a third independent coder. Since researchers tend to treat functioning as a secondary outcome, it was expected that some studies that included a relevant measure would not mention functioning in the abstract. This presented a unique challenge when it came to evaluating abstracts for eligibility. The workable solution was to assign a unique identifier to papers that looked to meet all the criteria except for evidence of a functioning outcome. At the conclusion of the abstract phase, the methods section of all the papers with that unique identifier (*n* = 70) was checked to see if a measure of functioning was included (*n* = 22). Next, the full text of studies that matched the inclusion criteria was downloaded and reviewed for eligibility by two independent coders, with a third independent coder acting as an arbiter if necessary. If there were multiple reports that used the same sample, it was decided to include the report that addressed the most functional domains; if both reports addressed the same number of functioning domains, the most recently published report would be included, so that the review focused on each study rather than each report.

### Data Collection Process

To assist in the data collection process, a data extraction form was developed using Microsoft Excel. A sample of five randomly selected studies was used to test and review the first draft of the data extraction form, which was then revised and finalised. Two independent coders extracted data from each eligible study. The most common source of inconsistency was an error made by one of the extractors, which was quickly resolved through discussion.

### Data Items

#### Outcome Measures for Which Data was Sought

Outcomes were improvement in the follow domains: (1) global functioning, defined as a single, composite rating of an individual’s ability to function psychosocially; (2) social functioning, defined as the ability to engage in and maintain social activities and perform social tasks; (3) academic performance, defined as the extent to which an individual is achieving or displaying the capacity to achieve educational goals; (4) school attendance, defined as presence at school; and (5) family functioning, defined as the dynamic of interpersonal interactions taking place amongst members of a family unit.

Studies were eligible if they met the inclusion criteria and reported data on at least one of the domains of functioning (above) at pre- and post-treatment. Post-treatment data were analysed from the assessment administered immediately after treatment (or the assessment administered closest to the end of treatment if there were multiple time points). Any measure of functioning that generated scores on continuous scales was eligible for inclusion. Data were entered as a scale with a consistent direction of effect (higher scores indicating improved functioning). Each study’s measures should have had evidence of validity and reliability for domain assessment; however, studies were not excluded on this basis. As it was expected that different instruments would be used to measure the same functional domains, data were analysed as standardised mean difference (SMD) effect sizes (Cohen’s d) and their 95% confidence intervals (CIs). In keeping with previous reviews, if multiple measures of the same domain were utilised, the measure that was the most common in the analysis was used to maximise consistency across studies (James et al., 2020). Where there were at least three studies contributing to the comparison, outcomes were entered and analysed separately by type of reporter (parent, child, and clinician). When scores were reported individually for mother and father, mother’s score was prioritised to maximise consistency across studies (Creswell et al., 2021). Outcomes could be reported as a single score that provided a composite measure across multiple domains of functioning, or as subscales that provided a measure of domain-specific functioning (e.g. Child Behaviour Checklist—Social Competence; Achenbach & Rescorla, 2001), or both. When the numerical data needed for the review was only presented in figures, the data were extracted using ‘WebPlotDigitalizer’ software Version 4.5 (Rohatgi, 2021).

#### Other Variables for which Data were Sought

The Report: Author, year, country of origin.

Participants: Sample size, mean, and standard deviation of age by experimental group, age range, %female by experimental group, primary anxiety diagnosis, or OCD.

The mean age, standard deviation of age, and percentage of females were recorded for each experimental group. If the information was not given for each experimental group, the overall mean age, standard deviation of age, and %female were entered. In keeping with previous reviews (Reynolds et al., 2012), studies where participants were aged between 7 and 12 years were classified as ‘child’, studies where the participants were aged between 13 and 17 years were classified as ‘adolescent’, and studies which included participants aged ± 12 years were classified as ‘mixed’.

### Intervention

#### Type of Psychotherapy

Psychotherapy was classified into three categories: CBT, Internet Cognitive Behaviour Therapy (ICBT), and others. CBT was defined as treatment that incorporated behavioural and cognitive models of learning: cognitive restructuring, psychoeducation, exposure (structured in vivo, imaginal), operant conditioning, and relaxation. ICBT was defined as CBT where treatment materials were delivered via information technology. ‘Other’ psychotherapies were defined as freestanding treatments that were not part of a wider CBT treatment package.

#### Type of Comparison Group

Comparison groups were primarily defined using Mohr et al., (2009) concepts and then refined by the coding team based on inspection of the control treatments used in qualifying studies. Psychotherapy was compared to (1) waitlist, which provided no intervention during the experimental treatment; (2) treatment-as-usual (TAU), which provided the routine non-study care ordinarily provided by the settings in which they were recruited; and (3) placebo (either pill placebo or therapy placebo), as determined by the original paper authors. Studies that compared two or more active treatments where all were viewed as equal (e.g. two psychotherapy treatments) and none of which was specified as the control condition were excluded. Where multiple treatment arms (e.g. psychotherapy versus medication versus control) were reported in the same study, only the relevant arms were examined. Studies with multiple treatment arms pose an analytical challenge for pair-wise meta-analysis (Borenstein et al., 2009). When two or more relevant treatments were compared to controls (e.g. CBT parent involved vs CBT parent not involved vs waitlist), data from the intervention that had been shown to be superior or were theoretically expected to be superior was used.

#### Delivery Format

Treatment delivery format was classified as either group or individual. Treatments classified as ‘individual’ were those in which a single young person was the focus of the treatment. Treatments classified as ‘group’ were those in which more than one young person was the focus of the treatment. When there was a combination of individual and group sessions, the prevailing format, that is, the format that consumed the majority of sessions, was used for classification.

#### Therapy Intensity

Therapy intensity was classified as either traditional therapy or low-intensity therapy. Traditional therapy included face-to-face interventions where the therapist had direct contact with the child or parent alone or with the child and the parent together. Low-intensity therapy included self-help/e-therapy/abridged versions of full CBT protocols that require minimal therapeutic input. When there was a combination of traditional and low-intensity sessions, the prevailing mode of therapy intensity, that is, the mode that consumed the majority of sessions, was used for classification.

### Study Risk of Bias Assessment

The Cochrane Collaboration’s tool for assessing risk of bias in RCTs was used as the basis for decisions on the risk of bias for included studies (Higgins et al., 2011). Two coders independently assessed the risk of bias in each study, and discrepancies were resolved through discussion where necessary. The tool comprises six specific categories of bias: sequence generation, allocation concealment, blinding, incomplete outcome data, selective outcome reporting, and other sources of bias. Each potential source of bias was judged by responding to a pre-determined question concerning the risk of bias for the category, for example, “Was the allocation sequence adequately generated?” (“yes” = low bias, “no” = high bias, “unclear” = unclear or unknown bias) and providing a justification for that judgement, e.g. “participants were randomly allocated”. Following the Cochrane Collaboration’s recommendations for RoB 2, an overall “risk of bias” judgement was made for each bias category (Higgins et al., 2011).

### Synthesis Methods

Statistical analyses were conducted using Revman Version 5.4, developed by the Cochrane Collaboration (2020). Outcomes were entered and analysed separately according to the type of reporter (child, parent, and clinician). The inverse-variance method was used, and the between-study variance was calculated using Cochrane’s *I*^2^ index, which assessed whether the variance in effect sizes between studies (heterogeneity) may be due to sampling error (Borenstein et al., 2009). Thresholds for the interpretation of *I*^2^ were based on recommendations by the Cochrane Collaboration: might not be important (0–40%); may represent moderate heterogeneity (30–60%); may represent substantial heterogeneity (50–90%); and considerable heterogeneity (75–100%; Deeks et al., 2021). Subsequently, a random-effects model was used in the meta-analysis to weight each study, generate a forest plot, and draw conclusions about effect sizes.

The above-mentioned model was chosen because the analyses indicated that the true effect could vary from study to study. Effect sizes (ES) were fitted to the data using standardised mean differences (SMD; Cohen, 1988). The interpretation of SMDs was guided by the following criteria: small (*d* = 0.2), medium (*d* = 0.5), and large (*d* = 0.8; Cohen, 1988). In the current review, a positive ES indicates improved functioning. When a study used a measurement scale that assessed the outcome in the other direction (lower score indicating less impairment), the ES was multiplied by − 1. When only standard errors (SE) were supplied, standard deviations were calculated by multiplying the SE by the square root of the sample size. Where only the pre-treatment or only the post-treatment standard deviation was available, the missing standard deviation was substituted by the other, because it was assumed that the intervention had no effect on the variability of the outcome measure. Where the standard deviation of the mean difference was not provided, it is imputed using the equation below (and similarly for the control group).

$$SD_{{{\text{Echange}}}} \, = \,\sqrt {SD^{2} Epre\, + \,SD^{2} Epost\, - \,(2\, \times \,corr\, \times \,SDEpre\, \times \,SDEpost)} ,$$

where *E* denotes the experimental group, *pre* denotes pre-treatment, *post* denotes post-treatment, *corr* denotes a theory-driven correlation coefficient describing how similar the pre- and post-treatment measurements were across participants. Where studies did not report the pre- to post-treatment correlations, a conservative correlation of 0.7 was assumed in accordance with recent research (Kreuze et al., 2018). The results were robust to sensitivity analyses around this correlation using correlations of 0.3 and 0.8 (see Appendix B). One study examined relevant outcomes in children and adolescents with a primary diagnosis of OCD (Lenhard et al., 2016). Sensitivity analyses that excluded Lenhard et al. (2016) from comparisons showed consistent results with the primary meta-analysis, indicating support for the inclusion of participants with a primary diagnosis of OCD.

To visually assess for possible publication bias, funnel plots were generated using Stata version 16.1. Additionally, regression tests (Egger et al., 1997) were run to statistically examine the asymmetry of the funnel plot for potential bias. In the absence of bias, the funnel plot will be shaped like a symmetrical (inverted) funnel and the p-value of the intercept on Egger’s test will be equal to or above 0.1. For comparisons with fewer than ten studies, no tests for publication bias were conducted because the test’s power would be insufficient to distinguish between chance and actual asymmetry (Page et al., 2019). Subgroup analyses were conducted (by delivery format and therapy intensity) to investigate potential moderators of treatment impact and possible sources of statistical heterogeneity. To ensure the meaningfulness of subgroup analyses, they were only conducted when at least three studies provided data for each subgroup (James et al., 2020).

## Results

### Study Selection

**Fig. 1:**
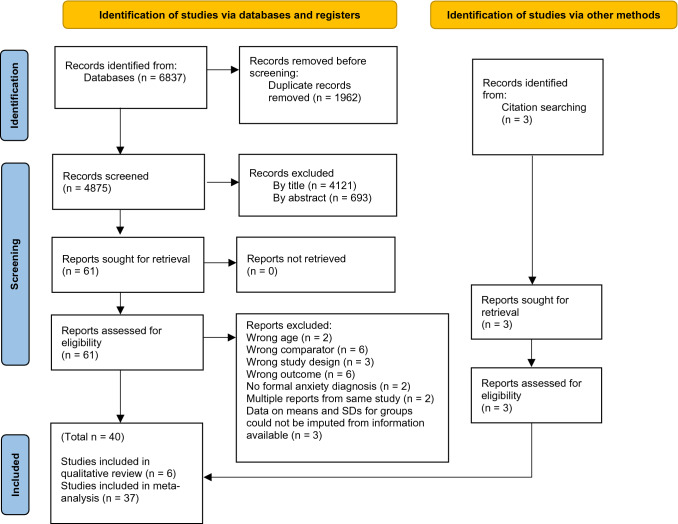
PRISMA flow diagram of literature search and study selection. From Page et al., (2021)

The method for identification, screening, and inclusion of studies is depicted in Fig. 1. The database search identified 6837 studies. 1962 duplicates were found and removed. Initial inter-rater agreement was moderate for the title stage (kappa 0.5), fair for the abstract phase (kappa 0.4), and almost perfect at full text (kappa 0.9). Of the 61 studies evaluated by full text, 24 did not meet the inclusion criteria. Of those, three appeared to meet all the inclusion criteria but were excluded due to insufficient data (Barrett et al., 1996; Cruz Pryor et al., 2021; Flatt & King, 2010). Later, the reference lists of reviews of similar topics were searched and three additional studies were found. As a result, the meta-analysis included 40 studies with a total of 3094 participants. Characteristics of the included studies are displayed in Appendix C.

### Risk of Bias in Studies

**Fig. 2:**
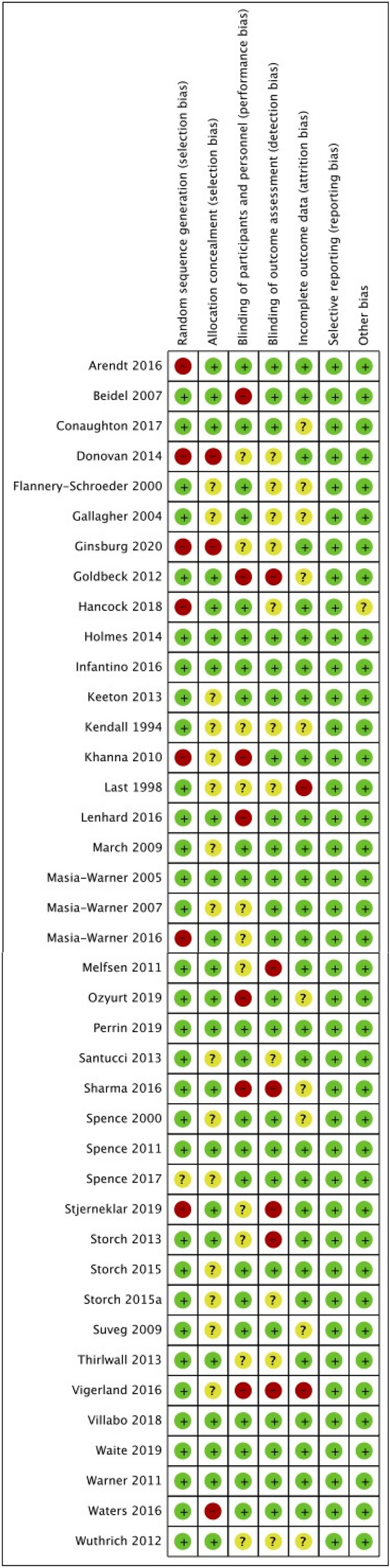
Risk of bias summary: review authors’ judgements about each risk of bias item for each included study

Each study’s risk of bias was assessed using the Cochrane RoB 2 for RCTs (Higgins et al., 2011). The majority of studies (32/40) highlighted concerns about risk of bias. The most frequently identified source of bias related to the blinding of key persons, which was not surprising given the challenges of blinding participants and treatment providers in psychological interventions. A summary of the risk of bias in the included studies is provided in Fig. 2.

### Results of Individual Studies

#### Global Functioning: Clinician Report

**Fig. 3:**
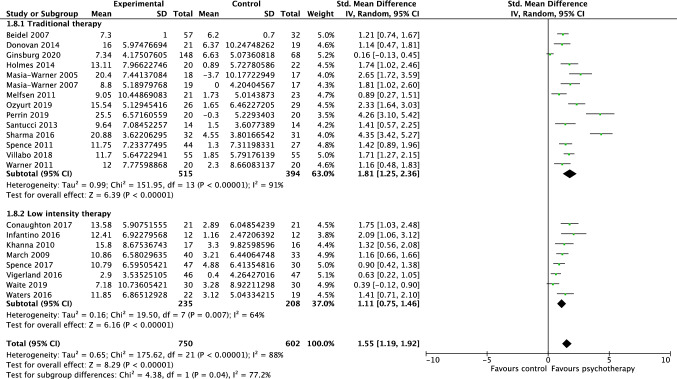
Forest plot of comparison: psychotherapy vs control, outcome: global functioning—clinician report by therapy intensity

**Fig. 4:**
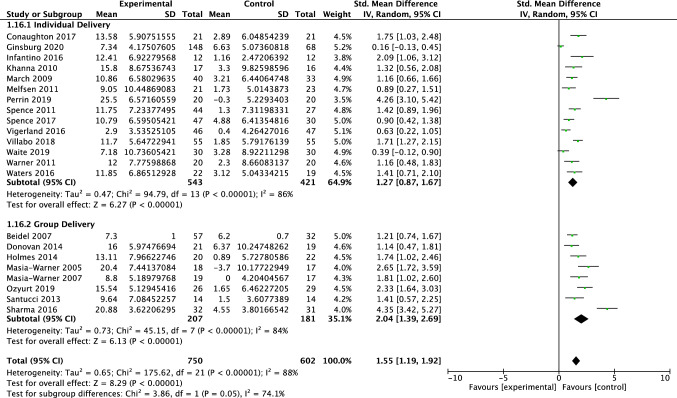
Forest plot of comparison: psychotherapy vs control, outcome: global functioning—clinician report by delivery format

Twenty-two studies, including 1352 participants, revealed a significant effect of psychotherapy on clinician-reported global functioning of 1.55 (95% CI = 1.19, 1.92; Z = 8.29 *p* < 0.001), with considerable heterogeneity shown by *I*^*2*^ of 88%. The test for subgroup differences in outcomes by therapy intensity indicated a statistically significant subgroup effect (*p* = 0.04; see Fig. 3). The treatment effect favoured psychotherapy for both the traditional and low-intensity therapy, although the treatment effect was greater for traditional therapy (*d* = 1.81) than for low-intensity therapy (*d* = 1.11). There was substantial unexplained heterogeneity between the studies within each of the subgroups (traditional = 91%; low intensity = 64%), indicating uncertain validity of the treatment effect estimate for each subgroup. The test for subgroup differences in outcomes for individual vs group therapy bordered significance (*p* = 0.05; see Fig. 4). The treatment effect favoured psychotherapy both group and individual delivery, although the treatment effect was greater for group delivery (*d* = 2.04) than for individual delivery (*d* = 1.27). There was considerable unexplained heterogeneity between the studies within each of the subgroups (individual = 86%; group = 84%), indicating uncertain validity of the treatment effect estimate for each subgroup.

#### Global Functioning: Parent Report

**Fig. 5:**
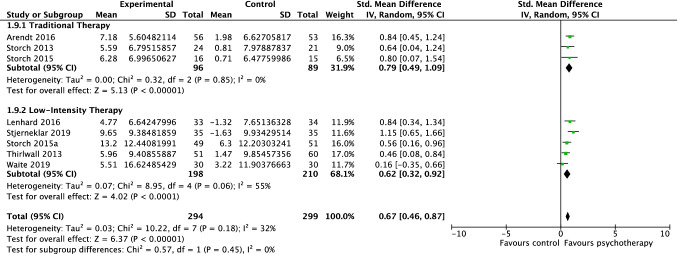
Forest plot of comparison: psychotherapy vs control, outcome: global functioning—parent report

Eight studies, including 593 participants, revealed a significant effect of psychotherapy on parent-reported global functioning of 0.67 (95% CI = 0.46, 0.87; Z = 6.37 *p* < 0.001), with moderate heterogeneity shown by *I*^2^ of 32%. The test for subgroup differences in outcomes for traditional vs low-intensity therapy indicated no statistically significant subgroup effect (*p* = 0.45; see Fig. 5). Due to the few studies evaluating group therapy (*n* = 1), no subgroup analysis was undertaken for delivery format.

#### Global Functioning: Child Report

**Fig. 6:**
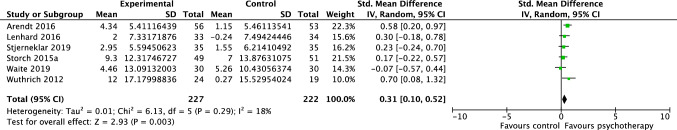
Forest plot of comparison: psychotherapy vs control, outcome: global functioning—child report

Six studies, including 449 participants, revealed a significant effect of psychotherapy on child-reported global functioning of 0.31 (95% CI = 0.10, 0.52; Z = 2.93 *p* = 0.003), with unimportant heterogeneity shown by *I*^2^ of 18% (see Fig. 6). Due to the uneven distribution of studies providing data to each of the planned subgroups, subgroup analysis would not have produced valid results.

#### Social Functioning: Parent Report

**Fig. 7:**
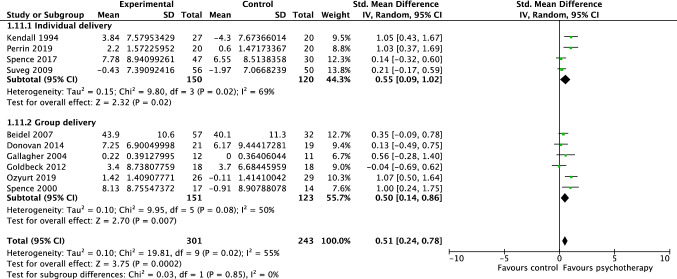
Forest plot of comparison: psychotherapy vs control, outcome: social functioning—parent report

Ten studies, including 544 participants, revealed a significant effect of psychotherapy on parent-reported social functioning of 0.51 (95% CI = 0.24, 0.78; Z = 3.75 *p* < 0.001), with substantial heterogeneity shown by *I*^2^ of 55%. The test for subgroup differences in outcomes by delivery format indicated no statistically significant subgroup effect (*p* = 0.85), suggesting that individual vs group delivery did not significantly modify the effect of psychotherapy when compared to controls (see Fig. 7). Due to the small number of studies evaluating social functioning following low-intensity therapy (*n* = 1), no subgroup analysis was undertaken for the comparison of traditional face-to-face therapy vs low-intensity therapy.

#### Social Functioning: Child Report

**Fig. 8:**
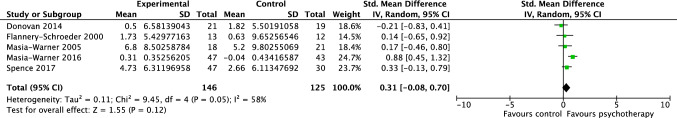
Forest plot of comparison: psychotherapy vs control, outcome: social functioning—child report

Five studies, including 271 participants, revealed a moderate but non-significant effect of psychotherapy on child-reported social functioning of 0.31 (95% CI = -0.08, 0.70; Z = 1.55 *p* = 0.12), with substantial heterogeneity shown by *I*^2^ of 58% (see Fig. 8). Due to the low number of studies providing data to this outcome, no subgroup analyses were undertaken.

### Publication Bias

For comparisons with a minimum of ten studies, funnel plots were used to visually assess for publication bias. Based on an examination of the asymmetrical funnel plot and the highly significant Egger’s test (*t* = 6.52, *p* ≤ 0.001), there was clear evidence of publication bias in the comparison of clinician-reported global functioning. Furthermore, a contour-enhanced funnel plot revealed a marked lack of non-significant studies, potentially indicating the existence of a file drawer effect. There was indication of asymmetry in the plot for parent-reported social functioning; however, the non-significant Egger’s test did not support this interpretation (*t* = − 0.69, *p* = 0.507). See Appendix D and E for funnel plots with contour lines corresponding to thresholds of statistical significance (*p* = 0.01, 0.05, 0.1).

## Discussion

Researchers examining the efficacy of anxiety treatments for children and adolescents have evaluated treatments based primarily on symptom or diagnostic improvements. However, focusing only on symptoms and diagnoses provides an incomplete picture of clinically meaningful efficacy, largely because impairment appears more important to consumers than symptoms (Creswell et al., 2021) and is a significant motivator for help-seeking (Sanchez et al., 2019). Thus the main objective of this study was to evaluate the overall impact of psychotherapy on global and domain-specific functioning in children and adolescents with anxiety disorders. Secondary objectives examined whether delivery format and therapy intensity moderated global and domain-specific functioning outcomes.

Global functioning following psychotherapy for anxiety in childhood and adolescence showed significant improvement and effects were large according to clinicians’ (*d* = 1.55), medium according to parents’ (*d* = 0.67), and young people’s (*d* = 0.31) reports. These effect sizes were comparable to those reported for symptom and diagnostic outcomes, which range between 0.87 and 1.36 for clinician report, 0.63 and 0.88 for parent report, and 0.36 and 0.77 for child report (Ishikawa et al., 2007; James et al., 2020; Kreuze et al., 2018; Perihan et al., 2020; Reynolds et al., 2012; Scaini et al., 2016; Wang et al., 2017). Our results, then, assessing functioning using a subjective, global, measure indicate that the benefits of anxiety-focused psychotherapy for children and adolescents extend beyond the treatment of symptoms and diagnoses to functioning across school, home, and with peers. Improvements on clinician-rated global functioning showed a surprisingly large effect. However, heterogeneity was large, particularly for clinician ratings, pointing to unreliability in the data as well as the likely importance of moderators. Nonetheless, the estimated effect on global functioning reported by clinicians remained considerably higher than that reported by parents (*d* = 0.67) or young people (*d* = 0.31). An earlier review that combined effects across raters (Kreuze et al., 2018) were unable to detect these informant differences and reported effects on global functioning that fall between the estimates in our review. The reasons for such large informant differences on global functioning are not clear and future studies may benefit from unpacking the bases upon which these different reporters make their ratings.

A novel focus in the current study was estimation of effects on specific domains of functioning. Unfortunately, one of the key results from this review was the dearth of clinical trials for treatment of child and adolescent anxiety that include measures of impact on specific domains of functioning. This is especially so for relatively objective outcome measures such as school absenteeism, grades, size of social networks, or frequency of social activities—variables that were almost unmeasured. Nonetheless, we were able to obtain limited estimates of effect size for one domain: social functioning.

Parent-reported social functioning demonstrated a significant treatment effect (*d* = 0.51); however, child reports showed a smaller effect that did not reach statistical significance (*d* = 0.31). This finding is congruent with existing literature on symptom-based outcomes in which larger treatment effects are frequently found for parent report than child report (e.g. Rapee et al., 2017). The only previous meta-analysis assessing the effect of CBT on social functioning (Kreuze et al., 2018) reported no significant effect of treatment but combined effects across parent and child report. By separating informants, the current study was able to provide more nuanced results.

Whilst we were theoretically capable of undertaking meta-analysis on family functioning, the fact that we identified only three papers constrained the evaluation to a small sample size that would have had to be accepted cautiously. As a result, it was determined that quantitative analysis would not add additional value. Rather, qualitative analysis was chosen as the best method for synthesis. Three studies examined changes in family functioning following psychotherapy for anxiety—each reporting moderate-to-large effect sizes in favour of psychotherapy (Arendt et al., 2016; Hancock et al., 2018; Keeton et al., 2013). Studies compared outcomes based on perceived family dysfunction attributed to having a child/adolescent with anxiety, including interference on parents’ own personal lives (Arendt et al. 2016; Hancock et al., 2018; Keeton et al., 2013). There was some indication that outcomes varied according to the type of reporter. Data from the Child and Adolescent Multimodal Study demonstrated that CBT resulted in reductions in family dysfunction according to child-, but not parent report (Keeton et al., 2013). In that study, youths who responded to CBT, compared to non-responders, were significantly more likely to demonstrate parent-reported improvement in family dysfunction, implying, at the very least, that the successful treatment of anxiety is related to improved family functioning (Keeton et al., 2013). These are promising treatment effects that merit replication.

Two RCTs reported on academic functioning, both failing to identify significant differences from pre- to post-treatment (Gallagher et al., 2004; Suveg et al., 2009). Studies measured academic functioning using the Child Behaviour Checklist (CBCL; Achenbach & Edelbrock, 1991), which assesses parental perception of children’s performance in academic areas. Frequent parent omission on CBCL items was noted and may have hindered the power to detect genuine differences between groups (Gallagher et al., 2004). Whilst participants in treatment reported similar improvement as controls from pre- to post-treatment (Gallagher et al., 2004; Suveg et al., 2009), there was some indication of a significant time effect where children who received CBT displayed significant improvements in academic functioning from post-treatment to follow-up, compared to attention controls (Suveg et al., 2009). It is possible that reduced anxiety following treatment may lead to a delayed academic benefit due to greater school attendance, improved school engagement, or improved attentional or general cognitive abilities. As a result, these effects may be detectable at follow-up but not at the time of post-treatment. This would be consistent with at least one study suggesting that reducing anxiety yields improvement in overall academic functioning (e.g. Wood, 2006).

For school attendance, a single study was identified for inclusion (Last et al., 1998). The purpose of the study was to examine changes in school attendance between an individualised CBT approach and an attention control condition consisting of educational presentations and supportive group psychotherapy. Average school attendance, measured by the school attendance record, increased significantly from pre- to post-treatment for CBT (40% increase) and the educational support condition (30% increase), but the difference between the two groups did not reach significance. Given the deleterious impacts of anxiety on academic performance and school attendance, a demonstrable benefit of treatment on school-related functioning is critical. Hence the lack of extensive evaluation of these constructs across the literature is surprising. One recent but currently unpublished study has shown that treatment of anxiety disorders in children with comorbid anxiety and attention-deficit hyperactivity disorder can lead to significant reductions in school absence (Sciberras et al., 2019).

Further, studies in the comparison of academic functioning used broad outcome measures based on parental perceptions of their child’s or adolescent’s performance in academic domains. Hence research is currently missing the opportunity to make use of readily available objective data from the school context, such as school attendance records or grades. Future treatment research should regard schools as a valuable source of objective data on this important area of functioning.

The considerably larger number of studies in our review compared to earlier reviews (James et al., 2020; Kreuze et al., 2018) allowed us to begin to examine two possible moderators of effects: delivery format (group vs individual) and therapy intensity (traditional vs low intensity). Unfortunately, even with the larger pool of studies, most subgroups were too small for analysis. Some hints began to emerge suggesting that group treatment delivery may produce slightly larger effects on functioning than individual delivery, at least for global functioning. This was an unusual finding, since most research on symptoms has indicated either that there are no consistent differences in outcomes according to delivery format (Ewing et al., 2015; Ishikawa et al., 2007; James et al., 2020) or that individual delivery occasionally has a stronger effect than group delivery (Reynolds et al., 2012). Given that this is the first time this effect has been demonstrated in the context of functioning, additional research is needed to assess its reliability. If the difference in efficacy between individual and group format proves to be robust to future research, it may suggest that characteristics of groups, such as increased motivation, group support, or opportunities for socialising, may have a unique beneficial impact on functioning.

We found evidence for statistically significant differences between the effects of traditional and low-intensity treatment based on clinician-reported global functioning. Parent-rated global functioning also showed 25% larger effects for participants who received traditional face-to-face therapy (*d* = 0.79) than participants who received low-intensity therapy (*d* = 0.62), but this difference was not statistically significant. This is inconsistent with effects from measures of symptom change, which have found few significant differences in outcome according to therapy intensity (James et al., 2020; Vigerland et al., 2016). Naturally, low-intensity interventions are delivered at considerably lower cost than traditional treatment and thus, even with a slightly lower efficacy these forms of intervention may strike a good cost-efficacy balance (Chatterton et al., 2017; Rapee et al., 2021). Nonetheless, future reviews of the impact of psychotherapy on functioning amongst anxious youth will need to carefully address this issue since it is possible that reducing therapist input to treatment might negatively affect its impact on measures of functioning.

The current review had two key strengths. First, study data were entered and analysed from multiple reporters separately, rather than integrating them, as past research on functioning had done. Whilst this meant that each analysis comprised only a subset of studies, the approach maximised the amount of captured information and minimised the variance owing to reporter discrepancies (Creswell et al., 2021). Hence the current review enriches understanding of functioning by representing multiple viewpoints. Future research on functioning should carefully consider the use of multiple and separate reporters. Secondly, it was anticipated that many study authors who included a measure of functioning might not have deemed it sufficiently important to reference in the abstract. It seems that the current perspective in efficacy trials that sees functioning as a secondary outcome posed a unique challenge for the meta-analytic search technique, which requires abstract screening to eliminate potential studies. By issuing a unique identifier to papers that appeared to match all the eligibility criteria except for reference to a functional domain and checking their methods, an additional twenty-two papers progressed to the full-text read stage. This additional step may have contributed to the greater number of studies included in the current review compared to the only previous review focused on functioning (Kreuze et al., 2018).

Although this study has noteworthy strengths, it also has some limitations. First, it was decided at the protocol stage not to contact study authors to clarify unclear information or gain access to unpublished results. It is possible that there were unpublished studies with relevant data that were not sought. Formal testing for publication bias indicated that this may be a limitation for global functioning but not for social functioning. Whilst every potentially eligible study was able to be accessed, three reports (Barrett et al., 1996; Flatt & King, 2010; Cruz Pryor et al., 2021) lacked the necessary data for the planned analysis and were excluded. As a result, the domain of family functioning was unable to be included. Given the clear associations between paediatric anxiety disorders and impairment in family life (Lawrence et al., 2015), assessing the impact of treatment on family functioning would have been valuable and remains a challenge for future research. Second, the search procedure identified a number of studies with school-refusing participants. Whilst a high proportion of school refusers meet the criteria for at least one anxiety disorder (McShane et al., 2001), studies that recruited participants for school refusal rather than for anxiety were excluded from the review because it was not always clear how many had diagnosable anxiety disorders. Anxiety problems are associated with generally poorer school attendance that may not “qualify” as school refusal (e.g. Lawrence et al., 2015). As a result, it was recognised that assessing school attendance as an outcome amongst broadly anxious children receiving treatment for anxiety (rather than school refusal) would address a substantial treatment gap. Despite an exhaustive search and scrutiny of over 4500 reports, only one such study was identified (Last et al., 1998), emphasising the need for researchers to include school attendance as a criterion against which to evaluate their anxiety treatments. In the literature more broadly, there is an overall lack of empirical research focused on functioning. James et al. (2020), for example, were able to identify eighty-seven face-to-face CBT trials providing data on the amelioration of anxiety symptoms and/or diagnoses in children and adolescents. By contrast, the current study, using a broader definition of psychotherapy, could identify just thirty-eight trials where changes in functioning were examined, and most of these used subjective reports of global functioning. Another limitation is related to the measurement precision of the field (Becker et al., 2011). The majority of studies included in this review employed broad and/or general measures of functioning, such as parental perceptions of how easily their child or adolescent makes friends. Only a few studies included objective outcome measures, such as the number of social activities per week. It is incumbent upon researchers to include well-defined and well-operationalised measures of functioning in the future trials in order to direct efforts towards the specific life impairments that anxiety treatments can reduce.

It is critically important that anxiety treatments reduce the personal costs associated with functional impairment, as well as the broader societal costs. In a recent Australian study on the population health costs associated with mental disorders in childhood and adolescence, anxiety disorders accounted for one of the highest expenditures (Le et al., 2021). If functional impairments play a greater role in bringing young people into services than symptoms and diagnoses (Sanchez et al., 2019), then these costs reflect how much money could be saved if anxiety treatments gave as much focus to functional improvements as they do to symptom and diagnostic improvements. The costs of functional impairments, however, extend beyond the direct costs of service utilisation to the indirect costs of missed school days and parent’s lost work hours due to their child’s or adolescent’s anxiety. One study of clinically anxious young people indicated that parents’ productivity losses due to their child’s anxiety (23% of total costs) and school absenteeism (17% of total costs) were major contributors to the burden of disease (Bodden et al., 2008). Hence, future research should place more emphasis on the specific life impairments that anxiety treatments assist, as functional outcomes are more likely to demonstrate the societal value of anxiety treatments and provide a clear rationale for policy makers to invest in such treatments.

The results from this study indicate a potentially significant impact of anxiety-focused psychotherapy on children’s and adolescent’s functioning. This study, then, addresses a significant gap in the literature regarding the efficacy of psychotherapy for bringing about clinically meaningful change in functioning amongst children and adolescents with anxiety disorders. By broadening the definition of treatment success to encompass the restoration of prior functioning or the optimisation of current functioning, clinicians and families may make more informed decisions about which treatments are “evidence-based” for the outcomes that matter most to them. Whilst the current study’s results are promising, they should be interpreted cautiously due to the dearth of empirical research in this area, compared to that of symptom/diagnostic outcomes. Additional targeted studies utilising clear and well-operationalised measures of functioning are required to properly evaluate treatments targeting anxiety disorders in children and adolescents.

## Supplementary Information

Below is the link to the electronic supplementary material.

Supplementary file1 (DOCX 968 kb)

## Data Availability

For this paper is not applicable since as a meta-analysis, all data have come from published papers.

## Footnotes

1 For full electronic search strategies for all databases, including filters and limits used, please contact the corresponding author.
