# Supplementary material for: Impact of Psychotherapy for Children and Adolescents with Anxiety Disorders on Global and Domain-Specific Functioning: A Systematic Review and Meta-analysis
Source: Clin Child Fam Psychol Rev. 2022 Jul 7;25(4):720–36. doi: 10.1007/s10567-022-00402-7 (PMC9622529; doi:10.1007/s10567-022-00402-7)
Supplement: Supplementary file 1 — Supplementary file1 (DOCX 968 kb) [file 10567_2022_402_MOESM1_ESM.docx]

**Appendix A**

**Catalogue of Search Terms Used as the Basis of Search Strategies**

| Concept | Terms |
| --- | --- |
| Anxiety | Anxiety disorder, clinical anxiety; generalised anxiety disorder, specific phobia; social phobia; social anxiety disorder; agoraphobia; panic disorder; separation anxiety disorder; obsessive compulsive disorder |
| Age | Child; adolescent; teenager; youth; minor; paediatric; young; school aged |
| Psychotherapy | Psychotherapy; intervention; cognitive therapy; training group; treatment; technique; cognitive behaviour therapy; psychoeducation; counselling; cognitive technique; program; parent delivered; guardian delivered; child delivered; parent focused; guardian focused; child focused; e-therapy; exposure therapy; relaxation; mindfulness; CBT; CBGT; iCBT; cCBT; bCBT |
| Study Design | Controlled clinical trial; randomised controlled trial; randomisation; random allocation; random assignment; random control; random treatment; control trial; control study; single blind; double blind; single mask; double mask; clinical trial; quasi random; quasi experimental; group treatment |
| Global Functioning | Global functioning; functioning; adaptive functioning; impairment; life impairment; interference; quality of life; real life outcome; secondary outcome; life impact; Strengths and Difficulties Questionnaire; Child Behaviour Checklist; Children’s Global Assessment Scale; Global Assessment of Functioning; daily activities; daily routine; daily living; daily life; extracurricular; after-school activities; recreational activities; activities of daily living; sport participation; sport engagement; sport attendance; hobby; |
| Social Functioning | Socioemotional functioning; peer relations; friendship; peer interaction; friends; popular; bully; peer rejection; social functioning; social skills; social competence; social adjustment; social interaction; social difficulty; social problem; social issue; socially adaptive behaviour; social ability; interpersonal skills; interpersonal competence |
| Academic Performance | School performance; school achievement; educational attainment; cognitive ability; cognitive performance; scholastic achievement; scholastic accomplishment; grade point average; homework; grade |
| School Attendance | School attendance; school termination; dropout; absent; absenteeism; days missed |
| Family Functioning | Family functioning; family relations; family problems; family dynamic; family life; family environment; sibling relations, parent-child relations; mother-child relations; family satisfaction |

**Appendix B**

**Sensitivity Analyses: Using Correlation 0.3 and 0.8 to impute Standard Deviation of Change**

| Comparison | Correlation 0.3 | Correlation 0.8 |
| --- | --- | --- |
| Global Functioning – Clinician Report | *d* = 1.38, 95% CI = 1.00, 1.77, Z = 7.07, *p* < .001 | *d* = 2.08, 95% CI = 1.58, 2.59, Z = 8.06, *p* < .001 |
| Global Functioning – Parent Report | *d* = 0.43, 95% CI = 0.27, 0.60, Z = 5.20, *p* < .001 | *d* = 0.79, 95% CI = 0.53, 1.05, Z = 5.97, *p* < .001 |
| Global Functioning – Child Report | *d* = 0.25, 95% CI = 0.06, 0.43, Z = 2.60, *p* = .009 | *d* = 0.48, 95% CI = 0.16, 0.80, Z = 2.91, *p* = .004 |
| Social Functioning – Parent Report | *d* = 0.32, 95% CI = 0.14, 0.51, Z = 3.40, *p* < .001 | *d* = 0.60, 95% CI = 0.26, 0.94, Z = 3.41, *p* < .001 |
| Social Functioning – Child Report | *d* = 0.25, 95% CI = -0.00, 0.50, Z = 1.95, *p* = 0.05 | *d* = 0.35, 95% CI = -0.12, 0.82, Z = 1.47, *p* = .14 |
| School-related Functioning | *d* = 0.46, 95% CI = -0.15, 1.08, Z = 1.47, *p* = .14 | *d* = 0.93, 95% CI = -0.08, 1.94, Z = 1.81, *p* = .07 |

**Appendix C**

*Characteristics of Included Studies*

| ­ | Study | Population | | |  |  | Intervention | |  |  | Outcome | |  |
| --- | --- | --- | --- | --- | --- | --- | --- | --- | --- | --- | --- | --- | --- |
|  |  | Country | *N* | Primary diagnosis | Age range | %Female | Psychotherapy | Delivery Format | Therapy Intensity | Control | Functioning | Measure | Reporter |
| 1 | Arendt 2016 | DNK | 109 | SAD, GAD, SocAD, SP, OCD, PD, AG | 7 -16 | 57 | CBT | Group | Trad | WL | Global, Family | CALIS-P, CALIS-C,  CALIS-P Mother | Child, Parent |
| 2 | Beidel 2007 | USA | 89 | SocAD | 7 -17 | 46.7 | CBT | Group | Trad | Plac | Global, social | CGAS, LS, CBCL-social | Clin |
| 3 | Conaughton 2017 | AUS | 42 | SocAD, GAD, SP | 8 -12 | 14.3 | ICBT | Individual | Low | WL | Global | CGAS | Clin |
| 4 | Donovan 2014 | AUS | 40 | SocAD | 8 -17 | 62.5 | CBT | Group | Trad | WL | Social, Global | CGAS, SSQ-P, SSQ-C, SCPQ-P, SCPQ-C | Child, Parent, Clin |
| 5 | Flannery-Shroeder 2000 | USA | 37 | GAD, SAD, SocAD | 8 -14 | 50.5 | CBT | Group, Individual | F2F | WL | Social | LS, FM-F, SAS-P | Child, Parent |
| ­ | Study |  | Population | |  |  | Intervention | |  |  | Outcome | |  |
|  |  | Country | *N* | Primary diagnosis | Age range | %Female | Psychotherapy | Delivery Format | Therapy Intensity | Control | Functioning | Measure | Reporter |
| 6 | Gallagher 2004 | USA | 23 | SocAD | 8 -11 | 52.17 | CBT | Group | Trad | WL | Social, Academic | CBCL-School, CBCL-Social | Parent |
| 7 | Ginsburg 2020 | USA | 216 | SAD, SocAD, GAD, SP | 6 -18 | 48.55 | CBT | Ind | Trad | TAU | Global | CGAS | Clinician |
| 8 | Goldbeck 2012 | DEU | 32 | SAD, GAD, SP, SocAD | 8 -12 | 50 | CBT | Group | Trad | TAU | Social | CBCL-Social withdrawal | Parent |
| 9 | Hancock 2018 | AUS | 157 | GAD, SocAD, SAD, OCD, AG, SP | 7-17 | 58 | CBT, Other | Group | Trad | WL | Family | CALIS-Family | Parent |
| 10 | Holmes 2014 | AUS | 42 | GAD | 7 -12 | 67.05 | CBT | Group | Trad | WL | Global | CGAS | Clinician |
| 11 | Infantino 2016 | AUS | 21 | SAD, SocAD, GAD, SP, OCD | 5 -11 | 54.17 | ICBT | Ind | Low | WL | Global | CGAS | Clinician |
| 12 | Keeton 2013 | USA | 215 | GAD, SocAD, SAD | 7-17 | 49.6 | CBT | Ind | Trad | Plac | Family | BFAM-III  BAS | Parent  Child |
| 13 | Kendall 1994 | USA | 42 | SAD | 9 -13 | 44 | CBT | Ind | Trad | WL | Social | CBCL-Social | Parent |
| ­ | Study |  | Population | |  | Intervention | |  | Outcome | |  |  |  |
|  |  | Country | *N* | Primary diagnosis | Age range | %Female | Psychotherapy | Delivery Format | Therapy Intensity | Control | Functioning | Measure | Reporter |
| 14 | Khanna 2010 | USA | 49 | SAD, SocAD, GAD, SP, PD | 7 – 13 | 32.65 | ICBT | Ind | Low | Plac | Global | CGAS | Clinician |
|  |  |  |  |  |  |  |  |  |  |  |  |  |  |
| 15 | Last 1998 | USA | 56 | SP | 6 -17 | 59 | CBT | Ind | Trad | Plac | Attendance | School Attendance record | Parent |
| 16 | Lenhard 2016 | SWE | 67 | OCD | 12 – 17 | 46.5 | ICBT | Ind | Low | WL | Global | EWSAS-P, EWSAS-C | Child, Parent |
| 17 | March 2009 | AUS | 73 | SAD, GAD, SocAD, SP | 7 -12 | 55.05 | ICBT | Ind | Low | WL | Global | CGAS | Clin |
| 18 | Masia-Warner 2005 | USA | 35 | SocAD | 13 – 17 | 74.2 | CBT | Group | Trad | WL | Social, Global | CGAS, LS | Child, Clin |
|  |  |  |  |  |  |  |  |  |  |  |  |  |  |
| 19 | Masia-Warner 2007 | USA | 36 | SocAD | 14 – 16 | 83.3 | CBT | Group | Trad | Plac | Global | CGAS | Clin |
|  |  |  |  |  |  |  |  |  |  |  |  |  |  |
| ­ | Study |  | Population | |  |  | Intervention | |  |  | Outcome | |  |
|  |  | Country | *N* | Primary diagnosis | Age range | %Female | Psychotherapy | Delivery Format | Therapy Intensity | Control | Functioning | Measure | Reporter |
| 20 | Masia-Warner 2016 | USA | 138 | SocAD | 14 – 16 | 68 | ICBT | Group | Trad | Plac | Social | SAS-SR | Child |
| 21 | Melfsen 2011 | DEU | 44 | SocAD | 8 -14 | 47.5 | CBT | Ind | Trad | WL | Global | CGAS | Clin |
|  |  |  |  |  |  |  |  |  |  |  |  |  |  |
| 22 | Ozyurt 2019 | TUR | 55 | SocAD, SP, SAD, GAD, PD | 8 – 12 | 36.47 | CBT | Group | Trad | WL | Social, Group | CGAS, SDQ-P-peer  SDQ-social | Parent, Clin |
| 23 | Perrin 2019 | UK | 40 | GAD | 10 – 18 | 62.5 | CBT | Ind | Trad | WL | Social, Global | CGAS, SDQ-P-peer | Parent, Clin |
| 24 | Santucci 2013 | USA | 28 | SAD | 7 -12 | 100 | CBT | Group | Trad | WL | Global | CGAS | Clin |
| 25 | Sharma 2016 | IND | 63 | SP, SocAD, PD, GAD, OCD, AG | 10 -19 | 47.65 | CBT | Group | Trad | TAU | Global | CGAS | Clin |
| 26 | Spence 2000 | AUS | 50 | SocAD | 7-14 | 32.8 | CBT | Group | Trad | WL | Social | SSQ-P  SCQ-P | Parent |
| ­ | Study |  | Population | |  | Intervention | |  | Outcome | |  |  |  |
|  |  | Country | *N* | Primary diagnosis | Age range | %Female | Psychotherapy | Delivery Format | Therapy Intensity | Control | Functioning | Measure | Reporter |
| 27 | Spence 2011 | AUS | 115 | GAD, SocAD, SAD, SP | 12-19 | 59.13 | CBT | Ind | Trad | WL | Global | CGAS |  |
| 28 | Spence 2017 | AUS | 125 | SocAD | 8 -17 | 60 | CBT | Ind | Low | WL | Global | CGAS | Clin |
| 29 | Stjerneklar 2019 | DNK | 70 | SocAD, GAD, SP, OCD, PD, SAD, AG | 13 – 17 | 78.5 | ICBT | Ind | Low | WL | Global | CALIS-P, CALIS-C | Parent |
| 30 | Storch 2013 | USA | 45 | SocAD, SAD, OCD, GAD | 7 – 11 | 19.9 | CBT | Ind | Trad | TAU | Global | CIS-P | Parent |
| 31 | Storch 2015 | USA | 31 | SocAD, SAD, OCD, GAD | 11 – 16 | 19.15 | CBT | Ind | Trad | TAU | Global | CIS-P | Parent |
| 32 | Storch 2015a | USA | 100 | SAD, SocAD, GAD, SP, PD | 7 – 13 | 44.05 | ICBT | Ind | Low | TAU | Global | CAIS-P  CAIS-C  CIS-P | Parent |
| ­ | Study |  | Population | |  |  | Intervention | |  |  | Outcome | |  |
|  |  | Country | *N* | Primary diagnosis | Age range | %Female | Psychotherapy | Delivery Format | Therapy Intensity | Control | Functioning | Measure | Reporter |
| 33 | Suveg 2009 | USA | 161 | GAD, SAD, SoCAD | 7 – 14 | 44 | ICBT | Ind | Trad | Plac | Social, Academic | CBCL-Social  CBCL-School | Parent |
| 34 | Thirlwall 2013 | UK | 194 | GAD, SocAD, SAD, PD, SP | 7 – 12 | 48.4 | CBT | Ind | Trad | WL | Global | CAIS-P | Parent |
| 35 | Vigerland 2016 | SWE | 93 | GAD, PD, SAD, SocAD, SP | 8 – 12 | 55.5 | ICBT | Ind | Low | WL | Global | CGAS | Clin |
| 36 | Villabo 2018 | NOR | 165 | SAD, SoCAD, GAD | 7 – 13 | 45.5 | CBT | Group, Ind | Trad | WL | Global | CGAS | Clin |
| 37 | Waite 2019 | UK | 60 | SocAD, GAD, SP, SAD, PD, AG | 13-18 | 65 | ICBT | Ind | Low | WL | Global | CGAS CAIS-P CAIS-C | Clin |
| 38 | Warner 2011 | USA | 40 | SAD, SocAD, GAD, SP | 8 – 16 | 65 | CBT | Ind | Trad | WL | Global | CGAS | Clin |
| ­ | Study |  | Population | |  |  | Intervention | |  |  | Outcome | |  |
|  |  | Country | *N* | Primary diagnosis | Age range | %Female | Psychotherapy | Delivery Format | Therapy Intensity | Control | Functioning | Measure | Reporter |
| 39 | Waters 2016 | AUS | 41 | SAD, GAD, SocAD, SP | 6 – 12 | 55.8 | Other | Ind | Low | WL | Global | CGAS | Clin |
| 40 | Wuthrich 2012 | AUS | 43 | GAD, SocAD, SAD, PD, OCD, SP | 14 – 17 | 62.30 | CBT | Ind | Low | WL | Global | ALIS | Child |

*Note.* Abbreviations: AUS (Australia); DNK (Denmark); USA (United States of America); IND (India); TUR (Turkey); UK (United Kingdom); DEU (Germany); SWE (Sweden); NOR (Norway); GAD (generalised anxiety disorder); SAD (separation anxiety disorder); SM (selective mutism); SocAD (social anxiety disorder); SP (specific phobia); OCD (obsessive-compulsive disorder); AG (agoraphobia); Ind (Individual); Trad (traditional therapy); Low (low intensity therapy); WL (waitlist); TAU (treatment-as-usual); Plac (placebo); CGAS (Children’s Global Assessment Scale); ALIS (Adolescent Life Interference Scale); CAIS-P/C (Child Anxiety Impact Scale – parent and child version); CBCL (Child Behavior Checklist – social/school subscales); CIS-P (Columbia Impairment Scale-Parent Version); CALIS-P/C/F (The Child Anxiety Life Inference Scale – parent/child/ family version); CALIS-P Mother – The Child Anxiety Life Interference Scale – interference on parent life; SSQ-P/C (The Social Skills Questionnaire- parent/child version); SDQ-P-peer (The Strengths and Difficulties Questionnaire – parent version – Peer Problems subscale); SAS-SR (Social Adjustment Scale–Self-Report – School subscales); LS (Loneliness Scale); EWSAS-P/C (The Education, Work and Social Adjustment Scale–Child and Parent Version); BFAM-III (The Brief Family Assessment Measure); BAS (Burden Assessment Scale); Clin (clinician)

**Appendix D**

**Funnel plot of Outcome: Global Functioning – Clinician report**

**
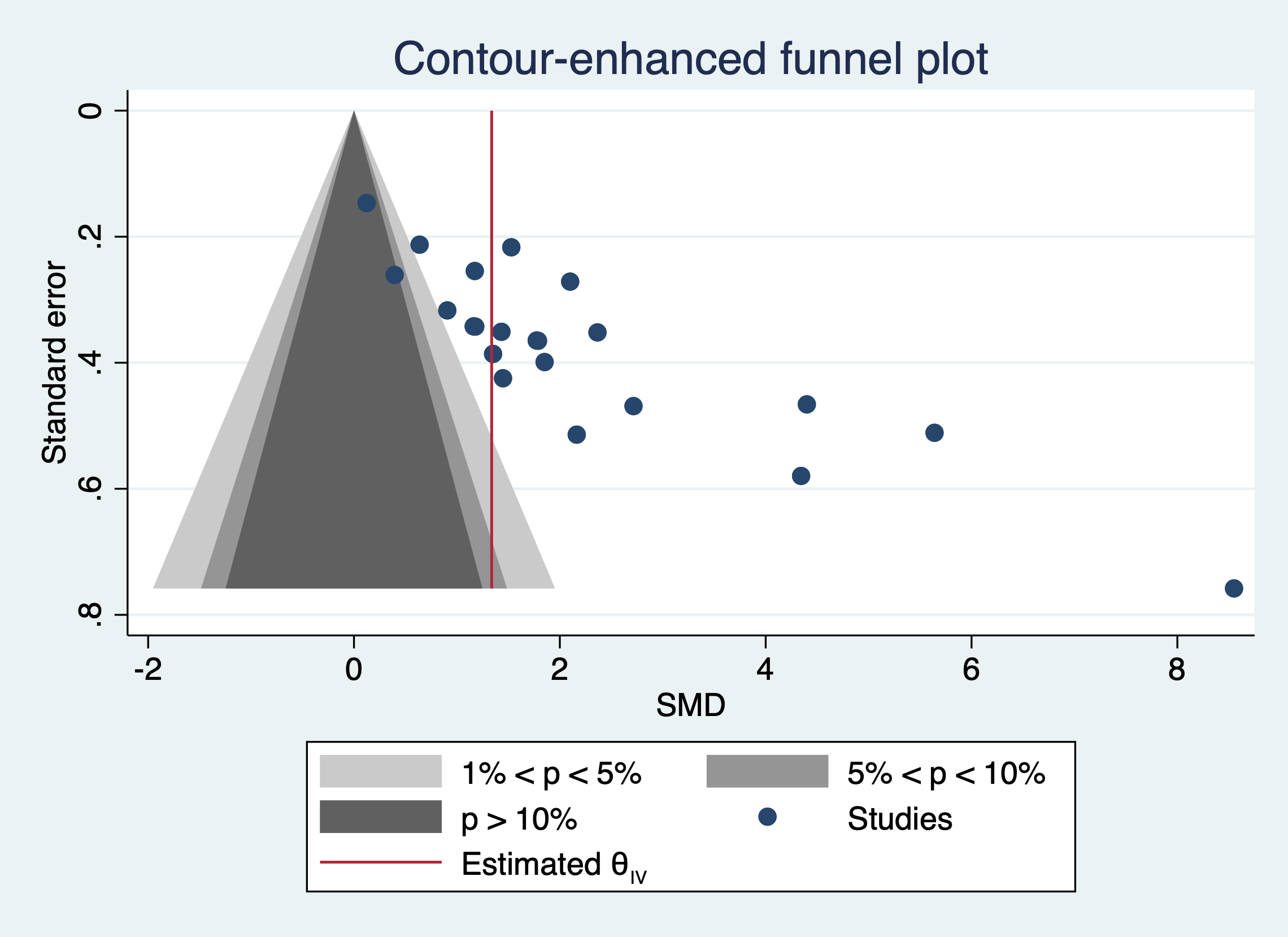
**

**Appendix E**

**Funnel plot of Outcome: Social Functioning – Parent report**

***
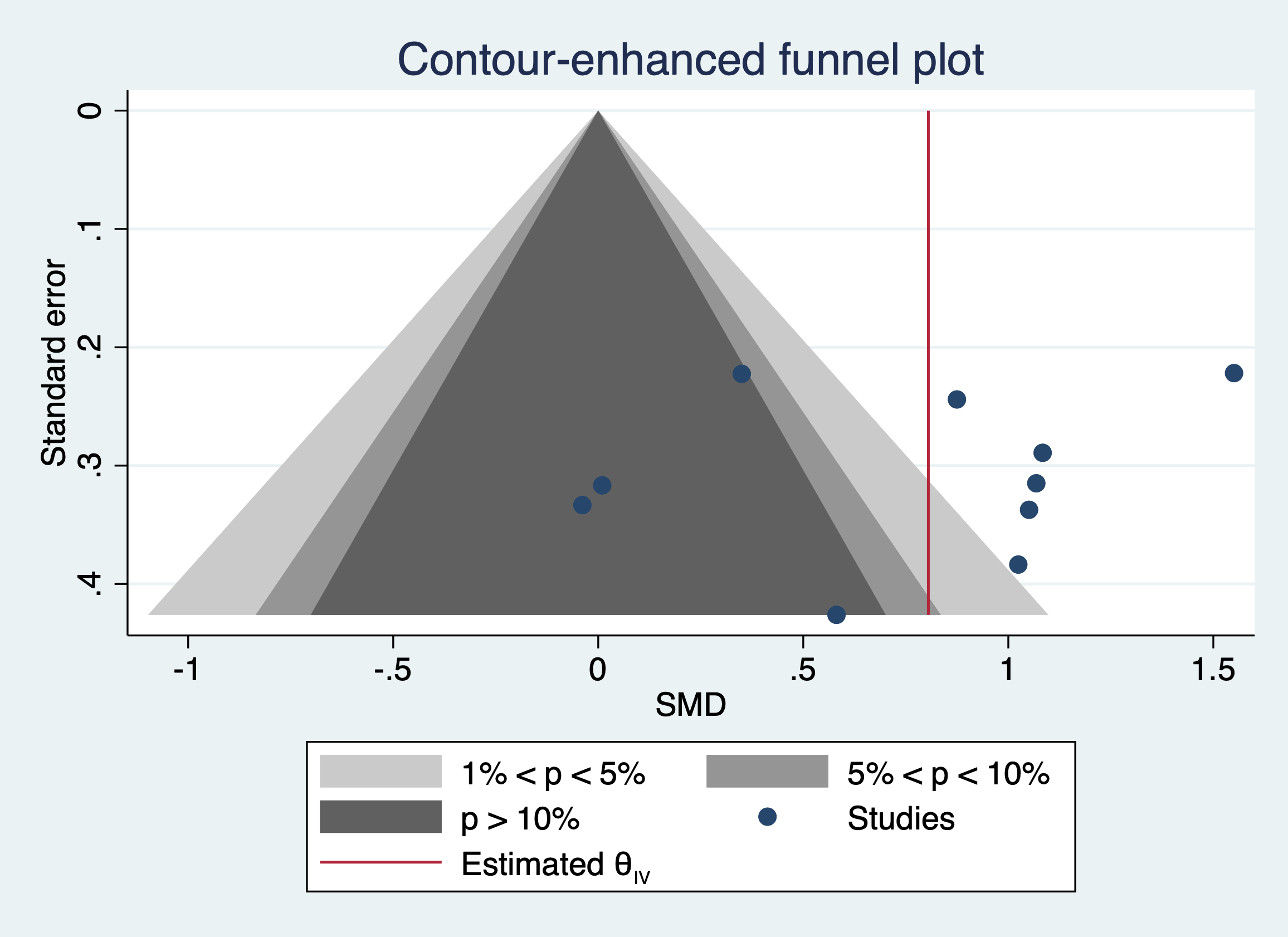
***

**Appendix F**

**References Cited in Systematic Review and/or Meta-analysis (not cited in text)**

Arendt, K., Thastum, M., & Hougaard, E. (2016). Efficacy of a Danish version of the Cool Kids program: a randomized wait-list controlled trial. *Acta Psychiatrica Scandinavica*, *133*(2), 109–121. https://doi.org/10.1111/acps.12448

Beidel, D. C., Turner, S. M., Sallee, F. R., Ammerman, R. T., Crosby, L. A., & Pathak, S. (2007). SET-C Versus Fluoxetine in the Treatment of Childhood Social Phobia. *Journal of the American Academy of Child and Adolescent Psychiatry*, *46*(12), 1622–1632. https://doi.org/10.1097/chi.0b013e318154bb57

Conaughton, R. J., Donovan, C. L., & March, S. (2017). Efficacy of an internet-based CBT program for children with comorbid High Functioning Autism Spectrum Disorder and anxiety: A randomised controlled trial. *Journal of Affective Disorders*, *218*, 260–268. https://doi.org/10.1016/j.jad.2017.04.032

Donovan, C. L., Cobham, V., Waters, A. M., & Occhipinti, S. (2014). Intensive Group-Based CBT for Child Social Phobia: A Pilot Study. *Behavior Therapy*, *46*(3), 350–364. https://doi.org/10.1016/j.beth.2014.12.005

Flannery-Schroeder, E. C., & Kendall, P. C. (2000). Group and Individual Cognitive-Behavioral Treatments for Youth with Anxiety Disorders: A Randomized Clinical Trial. *Cognitive Therapy and Research*, *24*(3), 251–278. https://doi.org/10.1023/A:1005500219286

Gallagher, H. M., Rabian, B. A., & McCloskey, M. S. (2004). A brief group cognitive-behavioral intervention for social phobia in childhood. *Journal of Anxiety Disorders*, *18*(4), 459–479. https://doi.org/10.1016/S0887-6185(03)00027-6

Ginsburg, G. S., Pella, J. E., Pikulski, P. J., Tein, J.-Y., & Drake, K. L. (2020). School-Based Treatment for Anxiety Research Study (STARS): a Randomized Controlled Effectiveness Trial. *Journal of Abnormal Child Psychology*, *48*(3), 407–417. https://doi.org/10.1007/s10802-019-00596-5

Goldbeck, L., & Ellerkamp, T. (2012). A Randomized Controlled Trial of Multimodal Music Therapy for Children with Anxiety Disorders. *The Journal of Music Therapy*, *49*(4), 395–413. https://doi.org/10.1093/jmt/49.4.395

Infantino, A., Donovan, C. L., & March, S. (2016). A randomized controlled trial of an audio-based treatment program for child anxiety disorders. *Behaviour Research and Therapy*, *79*, 35–45. <https://doi.org/10.1016/j.brat.2016.02.007>

Kendall, P. C. (1994). Treating Anxiety Disorders in Children: Results of a Randomized Clinical Trial. *Journal of Consulting and Clinical Psychology*, *62*(1), 100–110. https://doi.org/10.1037/0022-006X.62.1.100

Khanna, M. S., & Kendall, P. C. (2010). Computer-Assisted Cognitive Behavioral Therapy for Child Anxiety: Results of a Randomized Clinical Trial. *Journal of Consulting and Clinical Psychology*, *78*(5), 737–745. https://doi.org/10.1037/a0019739

Last, C. G., Hansen, C., & Franco, N. (1998). Cognitive‐Behavioral Treatment of School Phobia. *Journal of the American Academy of Child and Adolescent Psychiatry*, *37*(4), 404–411. https://doi.org/10.1097/00004583-199804000-00018

March, S., Spence, S. H., & Donovan, C. L. (2009). The Efficacy of an Internet-Based Cognitive-Behavioral Therapy Intervention for Child Anxiety Disorders. *Journal of Pediatric Psychology*, *34*(5), 474–487. https://doi.org/10.1093/jpepsy/jsn099

Masia Warner, C., Klein, R. G., Dent, H. C., Fisher, P. H., Alvir, J., Marie Albano, A., & Guardino, M. (2005). School-Based Intervention for Adolescents with Social Anxiety Disorder: Results of a Controlled Study. *Journal of Abnormal Child Psychology*, *33*(6), 707–722. https://doi.org/10.1007/s10802-005-7649-z

Masia Warner, C., Fisher, P. H., Shrout, P. E., Rathor, S., & Klein, R. G. (2007). Treating adolescents with social anxiety disorder in school: an attention control trial. *Journal of Child Psychology and Psychiatry*, *48*(7), 676–686. https://doi.org/10.1111/j.1469-7610.2007.01737.x

Masia Warner, C., Colognori, D., Brice, C., Herzig, K., Mufson, L., Lynch, C., Reiss, P. T., Petkova, E., Fox, J., Moceri, D. C., Ryan, J., & Klein, R. G. (2016). Can school counselors deliver cognitive-behavioral treatment for social anxiety effectively? A randomized controlled trial. *Journal of Child Psychology and Psychiatry*, *57*(11), 1229–1238. https://doi.org/10.1111/jcpp.12550

Melfsen, S., Kühnemund, M., Schwieger, J., Warnke, A., Stadler, C., Poustka, F., & Stangier, U. (2011). Cognitive behavioral therapy of socially phobic children focusing on cognition: A randomised wait-list control study. *Child and Adolescent Psychiatry and Mental Health*, *5*(1), 5–5. https://doi.org/10.1186/1753-2000-5-5

Ozyurt, G., Gencer, O., Ozturk, Y., & Ozbek, A. (2019). Is Triple P effective in childhood anxiety disorder? A randomized controlled study. *Psychiatry and Clinical Psychopharmacology*, *29*(4), 570–578. https://doi.org/10.1080/24750573.2018.1483790

Santucci, L. C., & Ehrenreich-May, J. (2013). A Randomized Controlled Trial of the Child Anxiety Multi-Day Program (CAMP) for Separation Anxiety Disorder. *Child Psychiatry and Human Development*, *44*(3), 439–451. https://doi.org/10.1007/s10578-012-0338-6

Sharma, P., Mehta, M., & Sagar, R. (2016). Efficacy of transdiagnostic cognitive-behavioral group therapy for anxiety disorders and headache in adolescents. *Journal of Anxiety Disorders*, *46*, 78–84. https://doi.org/10.1016/j.janxdis.2016.11.001

Spence, S., Donovan, C., & Brechman-Toussaint, M. (2000). The treatment of childhood social phobia: The effectiveness of a social skills training-based, cognitive-behavioural intervention, with and without parental involvement. *Journal of Child Psychology and Psychiatry*, *41*(6), 713–726. https://doi.org/10.1017/S0021963099005934

Spence, S. H., Donovan, C. L., March, S., Gamble, A., Anderson, R. E., Prosser, S., & Kenardy, J. (2011). A Randomized controlled trial of online versus clinic-based CBT for adolescent anxiety. *Journal of Consulting and Clinical Psychology,* 79(5), 629-642. https://doi.org/10.1037/a0024512

Spence, S. H., Donovan, C. L., March, S., Kenardy, J. A., & Hearn, C. S. (2017). Generic versus disorder specific cognitive behavior therapy for social anxiety disorder in youth: A randomized controlled trial using internet delivery. *Behaviour Research and Therapy*, *90*, 41–57. https://doi.org/10.1016/j.brat.2016.12.003

Stjerneklar, S., Hougaard, E., McLellan, L. F., & Thastum, M. (2019). A randomized controlled trial examining the efficacy of an internet-based cognitive behavioral therapy program for adolescents with anxiety disorders. *PloS One*, *14*(9), e0222485–e0222485. https://doi.org/10.1371/journal.pone.0222485

Storch, E. A., Arnold, E. B., Lewin, A. B., Nadeau, J. M., Jones, A. M., De Nadai, A. S., Jane Mutch, P., Selles, R. R., Ung, D., & Murphy, T. K. (2013). The Effect of Cognitive-Behavioral Therapy Versus Treatment as Usual for Anxiety in Children With Autism Spectrum Disorders: A Randomized, Controlled Trial. *Journal of the American Academy of Child and Adolescent Psychiatry*, *52*(2), 132–142.e2. https://doi.org/10.1016/j.jaac.2012.11.007

Storch, E. A., Lewin, A. B., Collier, A. B., Arnold, E., De Nadai, A. S., Dane, B. F., Nadeau, J. M., Mutch, P. J., & Murphy, T. K. (2015). A randomized controlled trial of cognitive‐behavioral therapy versus treatment as usual for adolescents with autism spectrum disorders and comorbid anxiety. Depression and Anxiety, 32(3), 174–181. https://doi.org/10.1002/da.22332

Storch, E. A., Salloum, A., King, M. A., Crawford, E. A., Andel, R., McBride, N. M., & Lewin, A. B. (2015a). A Randomized Controlled Trial in Community Mental Health Centers of Computer-Assisted Cognitive Behavioral Therapy Versus Treatment as Usual for Children with Anxiety. *Depression and anxiety*, *32*(11), 843–852. https://doi.org/10.1002/da.22399

Suveg, C., Hudson, J. L., Brewer, G., Flannery-Schroeder, E., Gosch, E., & Kendall, P. C. (2009). Cognitive-behavioral therapy for anxiety-disordered youth: secondary outcomes from a randomized clinical trial evaluating child and family modalities. Journal of Anxiety Disorders, 23, 341-349. https://doi.org/10.1016/j.janxdis.2009.01.003

Thirlwall, K., Cooper, P. J., Karalus, J., Voysey, M., Willetts, L., & Creswell, C. (2013). Treatment of child anxiety disorders via guided parent-delivered cognitive–behavioural therapy: Randomised controlled trial. *British Journal of Psychiatry*, *203*(6), 436–444. https://doi.org/10.1192/bjp.bp.113.126698

Vigerland, S., Ljótsson, B., Thulin, U., Öst, L.-G., Andersson, G., & Serlachius, E. (2016). Internet-delivered cognitive behavioural therapy for children with anxiety disorders: A randomised controlled trial. *Behaviour Research and Therapy*, *76*, 47–56. https://doi.org/10.1016/j.brat.2015.11.006

Villabø, M. A., Narayanan, M., Compton, S. N., Kendall, P. C., & Neumer, S.-P. (2018). Cognitive-Behavioral Therapy for Youth Anxiety: An Effectiveness Evaluation in Community Practice. *Journal of Consulting and Clinical Psychology*, *86*(9), 751–764. https://doi.org/10.1037/ccp0000326

Waite, P., Marshall, T., & Creswell, C. (2019). A randomized controlled trial of internet‐delivered cognitive behaviour therapy for adolescent anxiety disorders in a routine clinical care setting with and without parent sessions. *Child and Adolescent Mental Health*, *24*(3), 242–250. https://doi.org/10.1111/camh.12311

Warner, C. M., Colognori, D., Kim, R. E., Reigada, L. C., Klein, R. G., Browner-Elhanan, K. J., Saborsky, A., Petkova, E., Reiss, P., Chhabra, M., McFarlane-Ferreira, Y. B., Phoon, C. K., Pittman, N., & Benkov, K. (2011). Cognitive-behavioral treatment of persistent functional somatic complaints and pediatric anxiety: an initial controlled trial. *Depression and Anxiety*, *4*(2), 551–559. https://doi.org/10.1002/da.20821

Waters, A. M., Zimmer-Gembeck, M. J., Craske, M. G., Pine, D. S., Bradley, B. P., & Mogg, K. (2016). A Preliminary Evaluation of a Home-based, Computer-delivered Attention Training Treatment for Anxious Children Living in Regional Communities. *Journal of Experimental Psychopathology*, *7*(3), 511–527. https://doi.org/10.5127/jep.053315

Wuthrich, V. M., Rapee, R. M., Cunningham, M. J., Lyneham, H. J., Hudson, J. L., & Schniering, C. A. (2012). A randomized controlled trial of the Cool Teens CD-ROM computerized program for adolescent anxiety. *Journal of the American Academy of Child and Adolescent Psychiatry*, *51*(3), 261–270. https://doi.org/10.1016/j.jaac.2011.12.002
